# Determinants of Exposure to Fragranced Product Chemical Mixtures in a Sample of Twins

**DOI:** 10.3390/ijerph120201466

**Published:** 2015-01-27

**Authors:** Matthew O. Gribble, Karen Bandeen-Roche, Mary A. Fox

**Affiliations:** 1 Department of Preventive Medicine, University of Southern California Keck School of Medicine, Los Angeles, CA 90089, USA; 2 Department of Biostatistics, Johns Hopkins University Bloomberg School of Public Health, Baltimore, MD 21205, USA; E-Mail: kbandee1@jhu.edu; 3 Department of Health Policy and Management, Johns Hopkins University Bloomberg School of Public Health, Baltimore, MD 21205, USA; E-Mail: mfox9@jhu.edu

**Keywords:** fragrance, exposure assessment, risk perception, environmental psychology, galaxolide, HHCB, anosmia, exposure disparities, women’s health

## Abstract

Fragranced product chemical mixtures may be relevant for environmental health, but little is known about exposure. We analyzed results from an olfactory challenge with the synthetic musk fragrance 1,3,4,6,7,8-hexahydro-4,6,6,7,8,8-hexamethyl-cyclopento-γ-2-benzopyran (HHCB), and a questionnaire about attitudes toward chemical safety and use of fragranced products, in a sample of 140 white and 17 black twin pairs attending a festival in Ohio. Data for each product were analyzed using robust ordered logistic regressions with random intercepts for “twin pair” and “sharing address with twin”, and fixed effects for sex, age, education, and “ever being bothered by fragrances”. Due to the small number of black participants, models were restricted to white participants except when examining racial differences. Overall patterns of association were summarized across product-types through random-effects meta-analysis. Principal components analysis was used to summarize clustering of product use. The dominant axis of variability in fragranced product use was “more *vs.* less”, followed by a distinction between household cleaning products and personal care products. Overall, males used fragranced products less frequently than females (adjusted proportionate odds ratio 0.55, 95% confidence interval 0.33, 0.93). This disparity was driven by personal care products (0.42, 95% CI: 0.19, 0.96), rather than household cleaning products (0.79, 95% CI: 0.49, 1.25) and was particularly evident for body lotion (0.12, 95% CI: 0.05, 0.27). Overall usage differed by age (0.64, 95% CI: 0.43, 0.95) but only hand soap and shampoo products differed significantly. “Ever being bothered by fragrance” had no overall association (0.92, 95% CI: 0.65, 1.30) but was associated with laundry detergent use (0.46, 95% CI: 0.23, 0.93). Similarly, black *vs.* white differences on average were not significant (1.34, 95% CI: 0.55, 3.28) but there were apparent differences in use of shampoo (0.01, 95% CI: 0.00, 0.69), body lotion (4.67, 95% CI: 1.18, 18.47), and perfume (6.22, 95% CI:1.08, 35.89). There was no overall association with thinking about product risks (0.90, 95% CI: 0.79, 1.02), nor with inability to smell HHCB (0.84, 95% CI: 0.63, 1.12). Exposure to fragranced products may differ demographically. The relevance for health disparities should be studied.

## 1. Introduction

Fragranced product mixtures are a route of exposure to a variety of chemicals with possible health impacts [1]. Fragranced mixtures may contain diethyl phthalate [2], a possible weak aryl hydrocarbon receptor agonist [3]. In a sample of women from fertility clinics in Boston, lotion use was associated with higher urinary butyl-paraben, and perfume use with urinary monoethyl phthalate [4]. *In vivo* toxicological evidence suggests butyl-paraben might affect the uterus and male reproductive tract, and in an elderly Swedish cohort, a serum biomarker of monoethyl phthalate was associated with higher LDL cholesterol [5]. In some settings, cosmetics may also be a vehicle for exposure to nanoparticles [6]. The health importance of cumulative exposures to chemicals in fragranced consumer products is unknown.

Fragranced product mixtures often contain synthetic musk fragrances [7], but there are many other possible fragrance compounds including anise alcohol, amyl cinnama, benzyl alcohol, eugenol, limonene, methyl-2-octynoate, and others [8]. Synthetic musks have possible toxicological relevance as weak endocrine disruptors [9,10], and might act as dose-modifiers for xenobiotics, both through inhibition of broad-substrate transporters [11,12] and through modulation of cytochrome P450s [13,14]. We are unaware of epidemiologic research on polycyclic synthetic musk fragrances, and, excluding studies of dermatitis-associated musk ambrette [15], know of only one clinic-based case-comparison study of nitromusks [16], which suggested a possible association of musk xylene and musk ketone with gynecological dysfunction. However, there has been substantial literature on fragrance epidemiology more generally, in particular fragrance allergy contact dermatitis [17,18,19,20]. In a weighted survey of United States participants, 30.5% of the general population reported finding fragrances on others irritating, and 19.0% reported adverse health effects such as headaches and breathing difficulties from air fresheners or deodorizers; these symptoms were more frequent among persons with asthma, among whom 37.5% reported finding fragrances on others irritating and 33.5% reported having adverse health effects from air fresheners or deodorizers [21]. Exposure and toxicity of nitromusk exposures have been recently reviewed [22]. Usage of fragranced lotions and perfume was associated with blood musk levels in a sample of healthy young adults from Austria [23,24] indicating that product use, and consequent dermal exposure, is relevant for internal dose. In a comparison of older and younger women in Austria, older women had higher serum musk levels [25]. In Sweden, women with high use of perfume during pregnancy had elevated levels of the polycyclic musk fragrance 1,3,4,6,7,8-hexahydro-4,6,6,7,8,8-hexamethylcyclopento-γ-2-benzopyran (HHCB) in milk [26].

Synthetic musk fragrances are globally ubiquitous exposures, based on biomonitoring in the United States [27,28], South Korea [29,30], China [31], Italy [32], Denmark [33], and Germany [34,35]. A representative survey of the Flemish population detected the chemicals in 100% of samples, including umbilical cord blood [36,37]. Representative survey data from the United States are lacking, but California recently added synthetic polycyclic musks to its biomonitoring program’s designated chemicals list [38].

It is plausible that ability to smell the fragrance chemical HHCB may be related to fragranced product use or non-use. Products containing HHCB might lack a pleasant scent, or present offensive odors more strongly, to those who cannot perceive that fragrance. Since perception of the chemical HHCB has a strong genetic basis [39], this could be an example of genes influencing behaviors leading to fragranced product exposures, analogous to genes affecting smoking behaviors [40]. Other sensory perceptions might also be important for consumer choices.

Several studies have suggested that consumers’ attitudes toward health benefits from purchasing organic produce may influence their willingness to buy organic [41,42], but evidence is limited on the question of whether psychological attitudes toward chemical safety may influence consumer behaviors leading to exposure to fragranced product mixtures. A study of 250 Danish women associated attitudes with willingness to purchase cosmetics “free-of” certain toxic chemicals [43]. In Norway, cell phone software such as Forbrukerrådet Hormonsjekkare^®^ allows consumers to scan barcodes of products to check for endocrine-disrupting chemicals [44], and in Germany, the TOXFOX app serves a similar function [45]. A majority of Europeans do, or would, check the ingredients or composition of cosmetics/beauty products (59%) or cleaning products (53%) before buying [46]. Many Europeans would respond to a new product containing new chemical substances by waiting until it had been proven safe over a long period of time (46%) or buying only after finding sufficient information on its functionality and safety (30%). In contrast, a convenience sample survey of faculty, students and staff at a southeastern American university recruited on a day celebrating sustainability found that although health was the top-rated environmental concern, health concerns were not associated with willingness to pay more for an environmentally friendly product [47].

The main objective of this study was an exploratory analysis of several possible demographic, olfactory and attitudinal determinants of fragranced product use in a sample of twins ascertained in Ohio. We had also intended to examine heritability of attitudes in the twins, but not enough dizygotic twins were available for stable heritability estimates.

## 2. Materials and Methods

### 2.1. Ethics

This study was approved by the Penn IRB (Protocol #701426) and deemed exempt by the Johns Hopkins Bloomberg School of Public Health IRB. All participants provided informed consent.

### 2.2. Questionnaire Development

The study questionnaire (Questionnaire S1) was developed with two primary goals: to collect data on frequency of use of fragranced products, and to evaluate attitudes toward chemical safety. We selected specific fragranced products to inquire about usage on our questionnaire, based on a previous survey of polycyclic musk fragrance levels in United States consumer products [7] so that our questionnaire responses would act as a surrogate for exposure to mixtures containing polycyclic musk fragrances. Other questions, such as about familiarity with hazardous chemicals, were included to evaluate construct validity of the anticipated latent attitudes toward chemical safety [48]. We varied the order of answers (*i.e.*, Question 21 is ordered “Never or almost never” to “Almost Always” while Question 26 is ordered “Often” to “Never”). We also included an item with opposite directionality in the scale of attitudes toward chemical safety, expecting that the “belief that a store will sell only safe products’ should be disagreed with as participants agree with “thinking about the risks of products before buying’. We present results on the internal consistency, test-retest reliability, and criterion validity of the attitudinal scale vis-à-vis intended checks on construct validity.

### 2.3. Study Population

Participants for our study were recruited at the 2012 Twins Days festival in Twinsburg, OH, USA. To be eligible for our study, participants must have been over 18 years old, part of a twin pair, and both twins must have participated to be included. Most of the data, besides Galaxolide^®^ challenge results, come from the questionnaire developed for this study (Questionnaire S1). Some participants (*N* = 44) completed the questionnaire on a return visit and were used to assess within-weekend test-retest reliability of the questionnaire items.

For the HHCB challenge, a 5% v/v Galaxolide^®^ (formulation of HHCB) solution in mineral oil was prepared. Then 20 mL scintillation vials were filled with approximately 0.2 g of Viscopearls^®^ (Rengo, Co. LTD., Osaka, Japan—Viscopearls^®^ are cellulose beads, manufactured from wood pulp, that provide porosity and can provide gradual-release of the odorant), and 500 μl of the Galaxolide^®^ solution was added to the vial to be absorbed by the Viscopearls^®^, and capped. Subjects were instructed to uncap the vial, smell, and were then asked, “Did you smell something?” which was used to identify specific anosmia (“smell-blindness”) to the chemical HHCB. Follow-up questions among persons who could smell HHCB explored pleasantness and intensity ratings. We included odor detection, perceived pleasantness, and perceived intensity in the missing data imputation model, but only used HHCB detection in our main analyses.

Few participants self-identified with races other than black or white, so we excluded other racial groups from the analyses (*N* = 10) as well as participants who declined to identify with a race/ethnicity group (*N* = 6). The main analysis of fragranced product use was restricted to white participants only (*N* = 280). We included black race participants (*N* = 34) in secondary analyses to explore possible racial differences, but models were less stable after inclusion of race as a covariate. There were no differences between black and white participants’ attitudes toward chemical safety and therefore the two groups were pooled for summary description of those items’ responses (*N* = 314). Overall patterns of fragranced product use were examined in white participants who provided complete data regarding frequency of use of fragranced products (*N* = 242). The “N” reported are for clarity of identifying analysis populations (Table S1); the effective sample sizes are smaller because twins are not independent observations.

### 2.4. Data Quality Control

Questionnaire data were independently coded from paper questionnaire by two abstractors, and a consensus version was linked to demographic data from the taste genetics study. Invalid responses to the questionnaire (e.g., circling multiple answers or writing in words not corresponding to an answer) were treated as missing data. Missing data patterns were monotone among black or white participants. Data were inspected for within-person concordance in the test-retest sample, and for within-twin pair concordance for observations that should be the same within twin pairs (e.g., age and race/ethnicity; also sex for identical twins).

### 2.5. Statistical Methods

The test-retest reliability of each item was described using polychoric correlation and weighted kappa statistics. Polychoric correlation assesses agreement of two raters on scoring a latent variable, and thus is appropriate for assessing reliability of self-reported attitudes [49]. Kappa statistics are standard for test-retest reliability of categorical data; we used a weight equal to 1–|i–j|/(k–1), where i and j index the rows and columns of the possible ratings for each of the two visits, and k is the number of possible ratings [50]. Missing data for the questionnaire reliability and twin correlation analyses were handled by pairwise deletion. Dimensions of fragranced product use among white participants were examined by principal component analysis, with missing data handled by listwise deletion.

Regression analyses handled missing data by multiple imputation with chained equations [51] with 40 imputations. Frequencies of product use were binned into categories as needed for stable estimation (Table S1). Responses to questionnaire items on attitudes toward chemical safety were examined in pooled and twin-stratified samples, and twin similarities in attitudes were examined by polychoric correlations. Ordered logistic regression models with fixed effects for demographic determinants, olfactory perception, or concern for chemical safety (measured by the indicator “thinks of risks before buying products”), random effects for twin pair and sharing the same (reported) address, and robust standard errors were fit in Stata using generalized linear latent and mixed models (gllamm), with 12 adaptive quadrature points to approximate the loglikelihood numerically [52]. Random effects for zygosity were considered, but deemed unstable and not included in final models (not shown). We also considered associations with overall patterns of fragranced product use in mixed-effect linear regression models with principal component score as outcome, controlling for twin pair and living with twin, imputing missing data on covariates for the set of white participants with complete data on product use. All analyses were conducted in Stata SE 11.2.

We used random-effects meta-analysis to combine inferences across product types and thus get “broad brush-stroke” inferences about systematic differences in fragranced product mixture use that would be less susceptible to Type I error than individual comparisons for each product. Informed by our principal components analysis, we grouped “personal care products” and “cleaning products” for meta-analysis.

## 3. Results

### 3.1. Description of Study Sample and Measurement Properties of Instrument

Each questionnaire item (Questionnaire S1) had <5% missing data. The study samples for the main analysis (white only) and the analysis of fragranced product use were similar, but study samples for the main analysis and the reliability analysis differed in several respects (Table S1). The test-retest sample was more likely to report a shared address with twin, more likely to be identical twins, more likely to have post-high school education, more likely to be black, more likely to be female, less likely to prefer organic produce, and was on average slightly younger. Both samples were predominantly monozygotic twin pairs, reflecting the source population of the Twins Days Festival.

Most items had reasonable test-retest reliability. Self-report on fragranced product use had test-retest polychoric correlations ranging from 0.73 to 0.92, and weighted kappa statistics ranging from 0.52 to 0.88 (Table S2). The test-retest polychoric correlation for specific anosmia to HHCB was 0.61 (standard error 0.25), with kappa 0.34 (standard error 0.16) and observed agreement 79.41%. (There was a possible racial difference in the frequency of HHCB specific anosmia, with 29.4% of black participants and 17.8% of white participants reporting inability to smell HHCB.) There were 216 negative answers and 17 affirmative answers to having heard of synthetic musks. In the test-retest sample, only one participant indicated having heard of synthetic musks at the first visit, but did not repeat this answer on the second visit, so it is unclear how reliable positive self-report is. Thus, approximately 5% of the sample indicated familiarity with synthetic musk fragrances, but this may be an over-estimate if affirmative self-report is unreliable. Attitudes toward chemical safety as reported in our study are summarized in Table 1.

The majority of participants agreed that the public had a right to know what was in consumer products (98%), would stop using a product if it contained an unsafe ingredient (67%), agreed that products ought to be tested for safety (98%), and believed natural products were always safer (79%). Sibling polychoric correlations on measures of attitudes toward chemical safety were mild, ranging from 0.22 to 0.49. More detailed comparisons of sibling correlations are reported in Table S3.

The indicator “thinks about risks of products before buying” had variation in responses (Table 1) and was positively correlated with all other indicators of chemical concern yet negatively correlated with the indicator “trusts stuff at the store is safe” (Table S4). Thus, it was a reasonable measure of concern for chemical safety to use in regressions examining the relationship between concern for chemical safety and fragranced product use.

**Table 1:** Attitudes toward chemical safety. Black and white participants are pooled in this table (*N* = 314) because for these items responses did not differ significantly by race. Missing data were handled by pairwise deletion and reflected in the reported N per question.

		N (%)			
Item	Response	Pooled	Twin 1	Twin 2	Between-Twin Polychoric ρ (SE)
*“Natural fragrances are always safer than synthetic fragrances.”*	Strongly Disagree	5/310 (1.6%)	4/153 (2.6%)	1/157 (0.6%)	0.27 (0.11)
	Disagree	60/310 (19.4%)	38/153 (24.8%)	22/157 (14.0%)	
	Agree	165/310 (53.2%)	70/153 (45.8%)	95/157 (60.5%)	
	Strongly Agree	80/310 (25.8%)	41/153 (26.8%)	39/157 (24.8%)	
*“I believe the public has a right to know what specific chemicals are in products they use, such as shampoo, body lotion, perfume, laundry detergent, household cleaners, etc.”*	Strongly Disagree	3/311 (1.0%)	1/156 (0.6%)	2/155 (1.3%)	0.35 (0.10)
	Disagree	3/311 (1.0%)	3/156 (1.9%)	0/155 (<0.1%)	
	Agree	107/311 (34.4%)	52/156 (33.3%)	55/155 (35.5%)	
	Strongly Agree	198/311 (63.4%)	100/156 (64.1%)	98/155 (63.2%)	
*“Do you feel like you are able to find information about the safety of ingredients in products you use (shampoo, body lotion, perfume, laundry detergent, household cleaners, etc.)?”*	No, and I have no idea where I would find this information.	83/311 (26.7%)	43/156 (27.6%)	40/155 (25.8%)	0.36 (0.11)
	No, but I know someone who could help me find this information.	53/311 (17.0%)	20/156 (12.8%)	33/155 (21.3%)	
	Yes, I know where to find this information.	175/311 (56.3%)	93/156 (59.6%)	82/155 (52.9%)	
*“Would you stop using a product (such as shampoo, body lotion, perfume, laundry detergent, household cleaners, etc.) if you heard on the news that it contained something that might not be safe?”*	No	8/310 (2.6%)	4/156 (2.6%)	4/154 (2.6%)	0.22 (0.12)
	Maybe	95/310 (30.7%)	48/156 (30.8%)	47/154 (30.5%)	
	Yes	207/310 (66.8%)	104/156 (66.7%)	103/154 (66.9%)	
*“The products (shampoo, body lotion, perfume, laundry detergent, floor polish, etc.) a company sells to the public ought to be tested fully to be sure they are safe for everyone.”*	Strongly Disagree	1/308 (0.3%)	1/155 (0.7%)	0/153 (<0.1%)	0.41 (0.10)
	Disagree	5/308 (1.6%)	3/155 (1.9%)	2/153 (1.3%)	
	Agree	107/308 (34.7%)	50/155 (32.3%)	57/153 (37.3%)	
	Strongly Agree	195/308 (63.3%)	101/155 (65.2%)	94/153 (61.4%)	
*“How often do you read the labels on products (shampoo, body lotion, perfume, laundry detergent, household cleaners, etc.) you use?”*	Never or Almost Never	123/311 (39.6%)	56/157 (35.7%)	67/154 (43.5%)	0.46 (0.08)
	Sometimes	139/311 (44.7%)	73/157 (46.5%)	66/154 (42.9%)	
	Often	30/311 (9.7%)	15/157 (9.6%)	15/154 (9.7%)	
	Almost Always	19/311 (6.1%)	13/157 (8.3%)	6/154 (3.9%)	
*“How often do you think about the risks of the products you buy, before you buy them?”*	Never	51/307 (16.6%)	26/154 (16.9%)	25/153 (16.3%)	0.49 (0.08)
	Occasionally	75/307 (24.4%)	36/154 (23.4%)	39/153 (25.5%)	
	Sometimes	132/307 (43.0%)	63/154 (40.9%)	69/153 (45.1%)	
	Often	49/307 (16.0%)	29/154 (18.8%)	20/153 (13.1%)	
*“How often do you trust that a store will only sell safe products to use?”*	Rarely or Never	79/306 (25.8%)	37/153 (24.2%)	42/153 (27.5%)	0.24 (0.10)
	Sometimes	118/306 (38.6%)	57/153 (37.3%)	61/153 (39.9%)	
	Usually	109/306 (35.6%)	59/153 (38.6%)	50/153 (32.7%)	

### 3.2. Determinants of Use of Individual Fragranced Products

**Figure 1:**
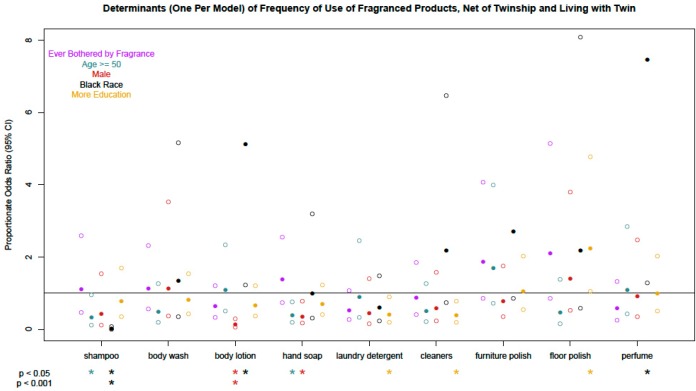
Determinants (one per model) of frequency of use of fragranced products, conditional on twinship and living with twin. Models are ordered logistic regressions with random intercepts for twin pair and address, fit separately for each demographic characteristic; all models except the model examining race (*N* = 314) were in white participants only (*N* = 280). Missing data were imputed by multiple imputation with chained equations. Filled circles are point estimates, empty circles are 95% confidence intervals. If confidence interval upper bound > 8, not shown. Asterisks below the abscissa flag associations as *p* < 0.05, or *p* < 0.001, without considering multiple comparisons.

Frequencies of use of individual fragranced product use are summarized in Table S5. Usage of fragranced products was significantly different according to demographics, conditional on twin pair and reporting a shared address with twin (Figure 1), and many of these associations persisted in multivariable-adjusted models (Figure S1).

In particular, males reported less frequent use of fragranced body lotion and blacks reported less frequent use of fragranced hair shampoo than female and white participants, respectively. Males also reported using fewer different fragranced products per week (crude POR 0.26, 95% CI: 0.13, 0.51; adjusted POR 0.25, 95% CI: 0.13, 0.49). Estimation for many of these multilevel ordered logistic models was imprecise, as reflected in large confidence intervals, and few were significant at *p* < 0.001. Associations of demographic characteristics with use of fragranced products attaining only moderate significance (0.001 ≤ *p* < 0.05) should be interpreted cautiously until replicated in future studies.

There were not significant differences in frequency of use of fragranced products according to HHCB specific anosmia among white participants (Table 2), conditional on twin pair and reporting a shared address with twin. 

**Table 2:** Associations of specific anosmia to the synthetic musk fragrance Galaxolide^®^ with frequency use of fragranced products among white participants (*N* = 280). Models are ordered logistic regressions with random intercepts for twin pair and address (Model 1) or with further adjustment for sex, age, education, and ever being bothered by fragrance (Model 2). Missing data were imputed by multiple imputation with chained equations.

Frequency of Use of Fragranced Products	Model 1	Model 2
Shampoo or conditioner	0.93 (0.27, 3.00)	1.06 (0.33, 3.37)
Shower soap or body wash	0.89 (0.36, 2.19)	0.98 (0.39, 2.45)
Hand soap	0.81 (0.34, 1.89)	0.94 (0.43, 2.05)
Body lotion	1.20 (0.54, 2.63)	1.06 (0.52, 2.15)
Perfume or cologne	1.14 (0.48, 2.69)	1.07 (0.46, 2.45)
Laundry detergent	0.54 (0.24, 1.21)	0.51 (0.24, 1.11)
Bathroom or kitchen cleaners	0.85 (0.36, 1.97)	0.78 (0.34, 1.78)
Floor polish	0.52 (0.18, 1.54)	0.59 (0.19, 1.78)
Furniture polish	0.76 (0.33, 1.74)	0.80 (0.34, 1.88)
# different fragranced products used per week	0.81 (0.38, 1.74)	0.78 (0.39, 1.54)

However, the point estimates for several products were < 1, with wide confidence intervals. The adjusted proportionate odds ratio for fragranced laundry detergent use was 0.51 (0.24, 1.11). The polychoric correlation between white twins for specific anosmia to HHCB was 0.42 (standard error 0.15), and kappa was 0.18 (standard error 0.06) with 84.5% agreement. 

“Thinking about risks” was not a significant predictor of fragranced product use (Table 3) although there was suggestively lower frequency of use of perfume (POR 0.72 95% CI: 0.48, 1.08) and perceived number of different fragranced products used per week (POR 0.72, 95% CI: 0.51, 1.02) in models adjusted for demographics and “being bothered” by fragrance, conditional on twin pair and reporting a shared address with twin.

**Table 3:** Associations of “thinking about risks” with frequency use of fragranced products and with exposure awareness among white participants (*N* = 280). Models are ordered logistic regressions with random intercepts for twin pair and address (Model 1) or with further adjustment for sex, age, education, and ever being bothered by fragrance * (Model 2). Missing data were imputed by multiple imputation with chained equations.

Frequency of Use of Fragranced Products	Model 1	Model 2
Shampoo or conditioner	0.92 (0.61, 1.39)	0.90 (0.59, 1.37)
Shower soap or body wash	1.08 (0.71, 1.64)	1.09 (0.70, 1.69)
Hand soap	0.94 (0.69, 1.28)	0.88 (0.64, 1.21)
Body lotion	1.23 (0.88, 1.72)	1.19 (0.85, 1.67)
Perfume or cologne	0.69 (0.47, 1.02)	0.71 (0.48, 1.06)
Laundry detergent	0.74 (0.49, 1.11)	0.76 (0.50, 1.15)
Bathroom or kitchen cleaners	0.83 (0.58, 1.17)	0.83 (0.58, 1.18)
Floor polish	0.87 (0.56, 1.34)	0.82 (0.53, 1.26)
Furniture polish	1.04 (0.71, 1.52)	0.96 (0.65, 1.41)
# different fragranced products used per week	0.79 (0.57, 1.11)	0.73 (0.52, 1.02)
**“Have you [ever] heard…”**		
“…that some plastic bottles may be unsafe to drink from, or heard of BPA (bisphenol A)?”	3.76 (1.88, 7.53)	4.85 (2.02, 11.67)
“…of synthetic musks like Galaxolide ®?” *	0.97 (0.41, 2.29)*	0.93 (0.41, 2.13)*
“…of lead, or thought about lead exposure?”	1.27 (0.83, 1.94)	1.27 (0.82, 1.95)
“…of mercury or thought about mercury exposure?”	1.83 (1.18, 2.81)	1.86 (1.20, 2.88)
“…of parabens or thought about exposure to parabens?”	3.18 (1.65, 6.14)	3.31 (1.66, 6.61)
Chemical Sensibilities		
Get bothered by fragrances **	1.74 (1.23, 2.44)	1.68 (1.19, 2.39)
Feel unwell after smelling fragrances in products **	1.70 (1.13, 2.57)	1.65 (1.08, 2.52)
Prefer to buy organic produce	3.15 (1.99, 4.96)	3.10 (1.97, 4.88)

* Affirmative self-report of Galaxolide^®^ familiarity may be unreliable. ** Model 2 for fragrance displeasure outcomes did not adjust for being bothered by fragrances.

### 3.3. Patterns of Use

The dimensions of variability in overall fragranced product use were explored by principal components analysis among white participants with complete data on product use (Table S6, Figure S2). The first principal component of fragranced product use had a positive loading for all products, distinguishing along the axis of “ever use” *versus* “never use” fragranced products. This is consistent with the common self-report of using multiple different fragranced products each week (Table S5). The second principal component (Table S6, Figure S2) distinguished fragranced products that were used for cosmetic or personal care purposes from products used for household cleaning. These two principal components explained 46% of the variability in fragranced product use. The third principal component was the last to have an eigenvalue approximately ≥1 (Table S6) and possibly suggests a distinction of fragranced laundry detergent and fragranced household cleaner use from use of the other products, with a greater similarity to the use of shampoo than other products. Visually examining the plots of these three principal components (Figure S2) it appears that patterns of fragranced product use vary widely across individuals. Among white participants with complete data on fragranced product use (*n* = 242), there was no association of the first principal component score (distinguishing more *vs.* less fragranced product use) with HHCB specific anosmia (−0.02, 95% CI −0.53, 0.49), “thinking about risks” (−0.03, 95% CI −0.24, 0.17), age (−0.39, 95% CI −0.98, 0.21), education (−0.24, 95% CI −0.64, 0.17), or ever being bothered by fragrance (0.03, 95% CI −0.40, 0.46), conditional on twin pair and living with twin. However, there was lower overall fragranced product use with male sex (−0.76, 95% CI −1.36, −0.16), conditional on twin pair and living with twin. 

### 3.4. Overall Differences in Use of Fragranced Products (Meta-Analysis)

**Figure 2:**
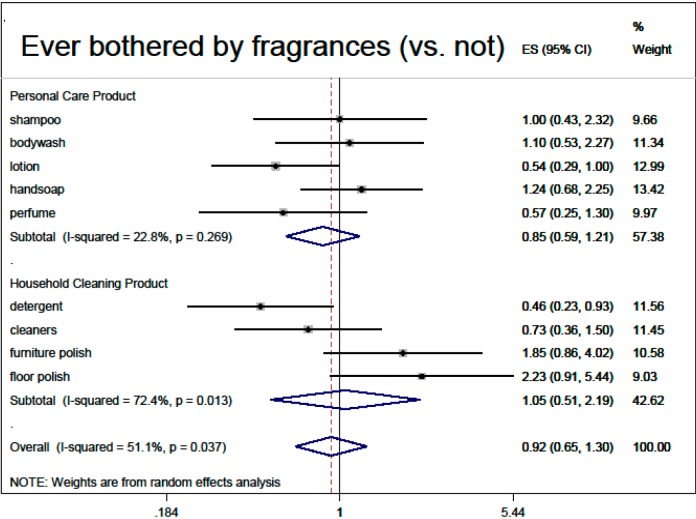
Demographic predictors of fragranced product use (meta-analysis of multivariable model results): ever bothered by fragrance (*vs.* not). product use (meta-analysis of multivariable model results): black race (*vs.* white). Models are ordered logistic regressions with random intercepts for twin pair and address, with fixed effects for age, sex, education, ever being bothered by fragrance and race, restricted to white participants only (*N* = 280). Missing data were imputed by multiple imputation with chained equations. Model estimates are combined by random-effects meta-analysis.

**Figure 3:**
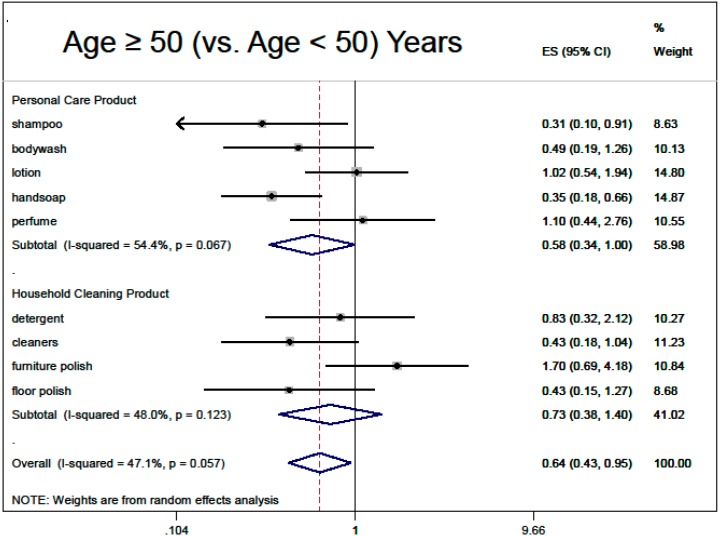
Demographic predictors of fragranced product use (meta-analysis of multivariable model results): age ≥ 50 (*vs.* younger). Models are ordered logistic regressions with random intercepts for twin pair and address, with fixed effects for age, sex, education, ever being bothered by fragrance and race, restricted to white participants only (*N* = 280). Missing data were imputed by multiple imputation with chained equations. Model estimates are combined by random-effects meta-analysis by product type.

**Figure 4:**
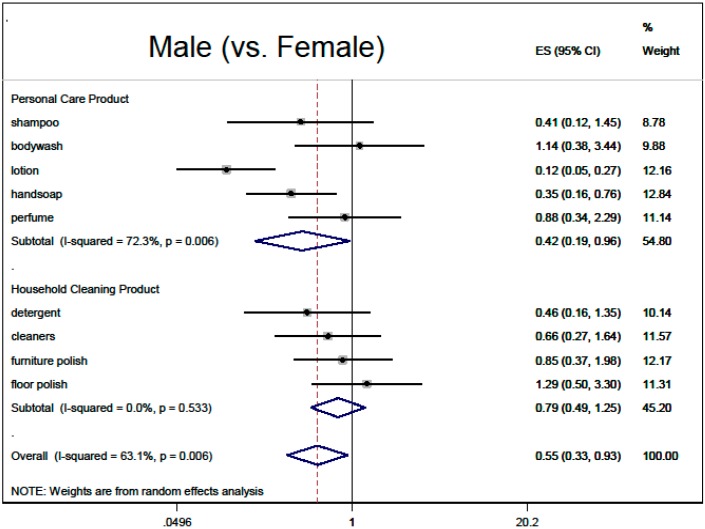
Demographic predictors of fragranced product use (meta-analysis of multivariable model results): male (*vs.* female). Models are ordered logistic regressions with random intercepts for twin pair and address, with fixed effects for age, sex, education, ever being bothered by fragrance and race, restricted to white participants only (*N* = 280). Missing data were imputed by multiple imputation with chained equations. Model estimates are combined by random-effects meta-analysis by product type.

**Figure 5:**
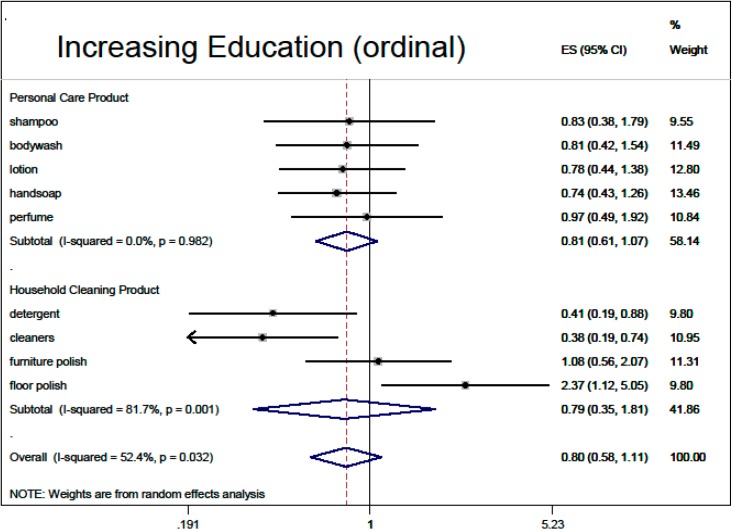
Demographic predictors of fragranced product use (meta-analysis of multivariable model results): increasing education (ordinal). Models are ordered logistic regressions with random intercepts for twin pair and address, with fixed effects for age, sex, education, ever being bothered by fragrance and race, restricted to white participants only (*N* = 280). Missing data were imputed by multiple imputation with chained equations. Model estimates are combined by random-effects meta-analysis by product type.

**Figure 6:**
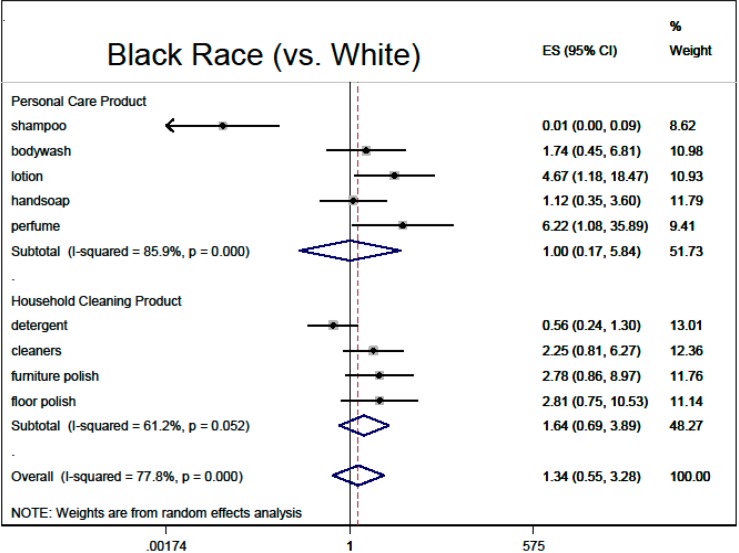
Demographic predictors of fragranced product use (meta-analysis of multivariable model results): black race (*vs.* white). Models are ordered logistic regressions with random intercepts for twin pair and address, with fixed effects for age, sex, education, ever being bothered by fragrance and race, among black and white participants (*N* = 314). Missing data were imputed by multiple imputation with chained equations. Model estimates are combined by random-effects meta-analysis by product type.

Based on the multivariable regressions, there was no overall association of fragranced product use with thinking about product risks (0.90, 95% CI: 0.79, 1.02), nor with inability to smell HHCB (0.84, 95% CI: 0.63, 1.12). Demographic adjusted associations with overall fragranced product use (“ever bothered by fragrance”, male sex, black race, older age, higher education) are summarized in meta-analysis Figure 2, Figure 3, Figure 4, Figure 5 and Figure 6.

## 4. Discussion

### 4.1. Main Findings

The most significant determinants of fragranced product use in this study were demographic variables. Males reported lower overall use, and in particular using fragranced body lotion less frequently. Racial differences were apparent in use of shampoo, body lotion and perfume. The tendency of consumers to use multiple fragranced products, illustrated in self-reported number of different fragranced products used per week (Table S5) and in the principal components analysis of frequency of use of each fragranced product, may be important for cumulative risk assessment. Although each consumer product may or may not be safe when considered independently, the aggregate intake of chemicals from multiple fragranced product mixtures should be evaluated in future studies.

We did not see significant associations between specific anosmia to HHCB and fragranced product use among white twins in regression models controlling for twin pair and living with twin. However, this should be interpreted cautiously because of the very high agreement between twins for specific anosmia to HHCB; we have a limited effective sample size for this analysis. We think the non-significant but potentially large magnitude association of specific anosmia to HHCB with less frequent use of fragranced laundry detergent bears further investigation in a different study population. Several point estimates for the proportionate odds ratios were smaller than one, with wide confidence intervals; we think it is important to consider these exploratory, non-significant but potentially large magnitude associations in future confirmatory studies, along with significant exploratory associations. Also, our study only evaluated one aspect of olfaction: ability to perceive HHCB. Other olfactory perceptions might also be relevant and could be explored in other studies.

We did not identify a relationship between attitudes toward chemical safety, as measured by the indicator “thinks about risks before buying products” and frequency of use of most fragranced products. In contrast, we did see significant associations of “thinking about risks” with familiarity with toxicants including mercury, parabens, and bisphenol A; with ever being bothered by fragrances or ever feeling unwell after smelling fragrances in products; and with preference for buying organic produce. These associations suggest that the scale item had construct validity, and that our study was able to detect strong associations of this variable with some strongly related constructs. However, it is possible that some of the non-significant, potentially large-magnitude associations in our study, such as for perfume use and perceived number of different fragranced products used each week, may have failed to attain significance only due to limited precision and might have been significant in a larger effective sample size study.

The substantial between-twin correlations in attitudes and behaviors suggest that heritability of these traits should be examined with additional data.

Also, we wish to highlight an unexpected finding: there was near-unanimous agreement in our sample to the statements “I believe the public has a right to know what specific chemicals are in products they use, such as shampoo, body lotion, perfume, laundry detergent, household cleaners, *etc.*” and “The products (shampoo, body lotion, perfume, laundry detergent, floor polish, *etc.*) a company sells to the public ought to be tested fully to be sure they are safe for everyone.” The strength of this opinion in our sample was surprising, but it is consistent with a recent survey of 1008 female undergraduates at Portland State University, which found that 92.5% agree or strongly agree that product labels should contain all ingredients, 87.6% agree or strongly agree that manufacturers should be responsible for testing all ingredients in personal care products for health impacts, and 91.1% agree that the ingredients in personal care products are personally important [53]. We think a nationally representative political opinion survey on attitudes toward chemical safety is warranted and could be informative for ongoing policy discussions.

There are many complex and interacting factors that can influence an individual’s choice to change behavior to reduce exposures to potential hazards (*i.e.*, to stop using a product after hearing it contained something that might not be safe), including prior risk perception, belief that the exposure can be controlled, voluntary nature of the exposure, and social context [54]. In the Portland State University undergraduate survey, there were negative associations between agreement with the statement “There are health risks associated with personal care products” and frequency of shampoo use, hair conditioner use, and shaving cream or gel use [53]. The Portland State University survey also had a non-monotonic association (significant by ANOVA) of perfume, cologne or body spray use with agreement with that statement: “never” and “monthly” users had highest agreement, followed by “daily” and “weekly”. In our sample, although we did not see any significant association between “thinking about risks” and fragranced product use, there was a suggestive association with perfume use (proportionate odds ratio 0.69, 95% CI: 0.47, 1.02), and 67% of our participants declared they would stop using a product if they heard on the news that it had a potentially unsafe ingredient.

There was not an association between most of the putative determinants with overall use of fragranced products as measured by the first principal component score. The only determinant that was significantly associated was sex, with males reporting less frequent use of fragranced products than females. This may be an artifact of multiple testing, but seems plausible and could be relevant for gender disparities in health outcomes.

### 4.2. Study Quality

Many participants reported the same address. Whether these same-given-address participants actually live together or are just providing the same address for study correspondence is not clear, but we think that the twins’ providing a common address is a possible indication of twin pairs that might be closer. 

The test-retest sample differed in several respects from the main study sample, and so there could be possible selection bias in generalizing from the test-retest subset. Although reliability in the test-retest study sample is encouraging, the performance of our questionnaire in another population may differ. 

We cannot assess the extent to which question order influenced the responses to the questionnaire, as all participants were administered the same version of the questionnaire. It is possible that prior questions may have influenced some of the responses to subsequent questions, and we asked about lead and mercury before probing participants’ attitudes toward chemical safety. If respondents were thus steered to express a higher concern on the attitudinal questions, this would introduce measurement error in their response and could reduce power to detect a true association of attitudes with behaviors. Therefore, there is a formal possibility that the observed null associations might be a technical artifact of the questionnaire as administered. However, it is also possible that the strength of individuals’ preferences might be much stronger than the cues (*i.e.*, perhaps these participants really do think that the public has a right to know the ingredients of products), in which case the change in responses induced by the question order would be modest. Future implementations of the questionnaire might consider adding questions with more positive connotations for fragranced products to offset some of the negative questions. 

This was a convenience sample of predominantly monozygotic twins with enough free time and discretionary income to be recruited at a festival in Ohio. This is an unusual group, so generalizing from our sample to a much broader population (*i.e.*, United States adults) may be inappropriate. It is possible that in a more representative sample of the general population, the performance of the questionnaire may differ, and attitudes and fragranced product use patterns could be different as well. Previous research suggests that black-white racial differences in consumer environmental concern may be weaker at higher income and education levels [55], suggesting our sample with high educational attainment and no observed racial difference in indicators of concern for chemical safety may not represent the general population. 

These preliminary findings are intriguing and worth following up in a more representative sample of the United States population. Although the measurement properties of our instrument would need to be confirmed, the performance of the instrument in our sample suggests that some of these questionnaire items could be reused in a national survey instrument.

Our study was limited to self-report questionnaire data for assessment of use of fragranced products. This might introduce some dependent measurement error into our estimates of the association between thinking about risks of products and use of various products, since all data were obtained from a single questionnaire. Future research using additional, complementary measures of fragranced product use, such as chemical exposure biomarkers, or store receipts, might furnish additional insights. 

## 5. Conclusions

This cross-sectional study found no statistically significant relationship between consumers’ thinking of the risks posed by products and their use of fragranced products, despite evidence that consumers who think about risks are more likely to prefer to purchase organic produce. While lack of significance could be due to sample size limitations, for many point-null associations it may suggest that fragrance chemicals’ ubiquity makes it difficult for motivated consumers to find fragrance-free alternatives; that consumer attitudes influence some exposure-related behaviors but not others; or that consumers are not aware of the possible risks of fragranced products they use. We think the latter possibility is plausible as only 5% of the sample had heard of synthetic musks. It has been suggested that even consumers with a higher concern for chemicals in food may not avoid phthalate exposures [56], and in one survey of fragrance contact allergy patients, 22% could not find a tolerable cosmetic product [57], so it is plausible that persons with high concern for chemical safety may not be able to limit their exposure to fragrances. We echo the recommendation by the National Academy of Science’s Committee on Human and Environmental Exposure Science in the 21st Century that “[i]nformation on chemical use and product ingredients also needs to be made publicly available to the greatest extent possible to allow the public to understand exposure pathways” [58] page 62, while also recognizing that merely listing ingredients on a label may be putting the onus of responsibility for safety on consumers who may be ill-equipped to read complex labels [59]. We observed demographic differences in use of specific fragranced products, and possibly differences in fragranced laundry detergent use according to HHCB anosmia which is partly genetically determined [39], and perfume use according to attitudes toward chemical safety. The relevance of these differential exposures for health disparities should be investigated in future studies.

## Supplementary Files

Supplementary File 1
