# Supplementary material for: Determinants of Exposure to Fragranced Product Chemical Mixtures in a Sample of Twins
Source: Int J Environ Res Public Health. 2015 Jan 27;12(2):1466–86. doi: 10.3390/ijerph120201466 (PMC4344677; doi:10.3390/ijerph120201466)
Supplement: Supplementary File 1 [file ijerph-12-01466-s001.pdf]

Supplementary Information

Determinants of Exposure to Fragranced Product Chemical Mixtures in a Sample of Twins

---

S1. Self-Administered Questionnaire Items

(1) How often do you use fragranced shampoo or conditioner?
- (a) More than once a day
- (b) Once a day
- (c) Several times a week
- (d) Once a week
- (e) Less than once a week
- (f) Never or almost never

(2) How often do you use fragranced shower soap or body wash?
- (a) More than once a day
- (b) Once a day
- (c) Several times a week
- (d) Once a week
- (e) Less than once a week
- (f) Never or almost never

(3) Have you ever heard of lead, or thought about lead exposure?
- (a) Yes
- (b) No

(4) How often do you use fragranced body lotion?
- (a) More than once a day
- (b) Once a day
- (c) Several times a week
- (d) Once a week
- (e) Less than once a week
- (f) Never or almost never

(5) How often do you use fragranced hand soap?
- (a) More than once a day
- (b) Once a day
- (c) Several times a week
- (d) Once a week
- (e) Less than once a week
- (f) Never or almost never

(6) Have you heard of synthetic musks like Galaxolide®?
- (a) Yes
- (b) No

(7) Have you ever heard of mercury or thought about mercury exposure?

(a) Yes
(b) No

(8) How strongly do you agree with the following: Natural fragrances are always safer than synthetic fragrances.

(a) Strongly agree
(b) Agree
(c) Disagree
(d) Strongly disagree

(9) How often do you use fragranced laundry detergent?

(a) More than once a day
(b) Once a day
(c) Several times a week
(d) Once a week
(e) Less than once a week
(f) Never or almost never

(10) How often do you use fragranced bathroom/kitchen cleaners?

(a) More than once a day
(b) Once a day
(c) Several times a week
(d) Once a week
(e) Less than once a week
(f) Never or almost never

(11) How often do you use fragranced furniture polish?

(a) More than once a day
(b) Once a day
(c) Several times a week
(d) Once a week
(e) Less than once a week
(f) Never or almost never

(12) How often do you use fragranced floor polish?

(a) More than once a day
(b) Once a day
(c) Several times a week
(d) Once a week
(e) Less than once a week
(f) Never or almost never

(13) How strongly do you agree with the following statement: "I believe the public has a right to know what specific chemicals are in products they use, such as shampoo, body lotion, perfume, laundry detergent, household cleaners, *etc*."

(a) Strongly agree

(b) Agree
(c) Disagree
(d) Strongly disagree

(14) How often do you wear perfume or cologne?
(a) More than once a day
(b) Once a day
(c) Several times a week
(d) Once a week
(e) Less than once a week
(f) Never or almost never

(15) Have you heard of parabens or thought about exposure to parabens?
(a) Yes
(b) No

(16) Do you feel like you are able to find information about the safety of ingredients in products you use (shampoo, body lotion, perfume, laundry detergent, household cleaners, *etc.*)?
(a) Yes, I know where to find this information.
(b) No, but I know someone who could help me find this information.
(c) No, and I have no idea where I would find this information.

(17) Do you get bothered by fragrances?
(a) Never
(b) Sometimes
(c) Often
(d) Almost always

(18) Would you stop using a product (such as shampoo, body lotion, perfume, laundry detergent, household cleaners, *etc.*) if you heard on the news that it contained something that might not be safe?
(a) Yes
(b) Maybe
(c) No

(19) How strongly do you agree with the following statement: "The products (shampoo, body lotion, perfume, laundry detergent, floor polish, *etc.*) a company sells to the public ought to be tested fully to be sure they are safe for everyone."
(a) Strongly agree
(b) Agree
(c) Disagree
(d) Strongly disagree

(20) How many different fragranced products (shampoo, body lotion, perfume, laundry detergent, household cleaners, air fresheners, *etc.*) do you think you use on average each week?
(a) None.
(b) 1 to 3 different products.
(c) 4 to 6 different products.

(d) 7 to 10 different products.
(e) More than 10 different products.
(f) I'm not sure.

(21) How often do you read the labels on products (shampoo, body lotion, perfume, laundry detergent, household cleaners, *etc.*) you use?
(a) Never or almost never
(b) Sometimes
(c) Often
(d) Almost always

(22) Do you ever feel unwell after smelling fragrances in products?
(a) Never
(b) Sometimes
(c) Often
(d) Almost always

(23) Do you ever feel unwell from smelling perfumes or colognes that other people wear?
(a) Never
(b) Sometimes
(c) Often
(d) Almost always

(24) Where do you use air fresheners? (Circle all that apply)
(a) Bathroom
(b) Laundry room
(c) Living room/den
(d) Bedroom
(e) Basement
(f) Car
(g) Never use air fresheners

(25) Have you ever heard that some plastic bottles may be unsafe to drink from, or heard of BPA (bisphenol A)?
(a) Yes
(b) No

(26) How often do you think about the risks of the products you buy, before you buy them?
(a) Often
(b) Sometimes
(c) Occasionally
(d) Never

(27) Do you prefer to buy organic produce?
(a) Yes
b) No

(28) Education Level (Circle closest description)

No high school
Some high school
High School Graduate
Some College
College Graduate
Post-Graduate

(29) Were you home-schooled for a length of time (at least one year after kindergarten)?
(a) Yes
(b) No

(30) How often do you trust that a store will only sell safe products to use?
(b) Usually
(c) Sometimes
(d) Rarely or Never

Table S1. Self-reported participant characteristics in the study samples, N (%). Percentages are out of reported data.

Twin-Level Characteristics	All Participants (1st Visit)	Main Analysis (1st Visit, White Only)	Test-Retest (1st visit)	Test-Retest (2nd visit)	Product Use(1st Visit, White Only, Listwise Deletion *)
Share (Reported) Address	118 (38%)	108 (39%)	20 (50%)	20 (50%)	97 (40.1%)
Monozygotic (Identical)	266 (86%)	234 (85%)	38 (95%)	38 (95%)	201 (83.1%)
Dizygotic (Fraternal)	42 (13%)	40 (14%)	2 (5%)	2 (5.0%)	35 (14.5%)
Unknown Zygosity by Self-Report	6 (2%)	6 (2%)	0 (0%)	0 (0%)	6 (2.5%)
Individual Characteristics					
Male	82 (26%)	76 (27%)	12 (30%)	12 (30.0%)	69 (29%)
Mean Age (SD)	39.5 (17)	38.1 (17)	33.3 (12.7)	33.3 (12.7)	37.7 (16.8)
Race/Ethnicity					
White	280 (89%)	280 (100%)	34 (85%)	34 (85%)	242 (100%)
Black	34 (11%)	0 (0%)	6 (15%)	6 (15%)	0 (0%)
Educational Attainment					
Some High School	6 (2%)	6 (2%)	0 (0%)	0 (0%)	5 (2%)
High School Graduate	53 (17%)	48 (18%)	5 (13%)	6 (15%)	38 (16%)
Some College	87 (28%)	73 (27%)	12 (31%)	13 (33%)	65 (28%)
College Graduate	114 (37%)	104 (38%)	14 (36%)	14 (35%)	91 (39%)
Postgraduate	47 (15%)	43 (16%)	8 (21%)	7 (18%)	37 (16%)
Prefer to Buy Organic	146 (48%)	136 (50%)	17 (44%)	18 (45%)	123 (52%)
Ever Bothered by	215 (69%)	194 (70%)	30 (75%)	28 (70%)	166 (69%)
Ever Unwell from	133 (43%)	121 (44%)	17 (43%)	21 (53%)	101 (42%)

* Listwise deletion based on use of fragranced product questions only.

Table S2. Questionnaire test-retest reliability in test-retest sample. Missing data handled by pairwise deletion. Reliability is for self-reported scale of observations, except # of fragranced products (recoded "I don't know" as missing).

Frequency of Use of Fragranced Products	Polychoric ρ (SE)	Weighted κ (SE)
Shampoo or conditioner	0.85 (0.07)	0.68 (0.12)
Shower soap or body wash	0.73 (0.13)	0.62 (0.11)
Hand soap	0.92 (0.04)	0.75 (0.12)
Body lotion	0.77 (0.08)	0.64 (0.12)
Perfume or cologne	0.96 (0.03)	0.88 (0.12)
Laundry detergent	0.84 (0.09)	0.76 (0.10)
Bathroom or kitchen cleaners	0.74 (0.12)	0.63 (0.10)
Floor polish	0.84 (0.07)	0.54 (0.11)
Furniture polish	0.82 (0.07)	0.52 (0.12)
# different fragranced products used per week	0.79 (0.10)	0.56 (0.12)
**Locations of Use of Air Fresheners**		
Use in bathroom	1.00 (< 0.01)	0.75 (0.15)
Use in bedroom	0.90 (0.08)	0.71 (0.16)
Use in laundry room	0.93 (0.06)	0.73 (0.16)
Use in living room / den	0.96 (0.04)	0.80 (0.16)
Use in basement	Not estimable	0.73 (0.15)
Use in car	0.99 (0.02)	0.89 (0.16)
Never use air fresheners	Not estimable	0.93 (0.16)
**"Have you [ever] heard…"**		
"…that some plastic bottles may be unsafe to drink from, or heard of BPA (bisphenol A)?"	0.95 (0.07)	0.77 (0.16)
"…of lead, or thought about lead exposure?"	0.83 (0.14)	0.53 (0.15)
"…of mercury or thought about mercury exposure?"	Not estimable	0.92 (0.16)
"…of parabens or thought about exposure to parabens?"	Not estimable	0.80 (0.16)
**Chemical Sensibilities**		
Get bothered by fragrances	0.96 (0.04)	0.69 (0.12)
Feel unwell after smelling fragrances in products	0.82 (0.08)	0.56 (0.13)
Prefer to buy organic produce	0.97 (0.03)	0.84 (0.16)
**Attitudes Concerning Chemical Safety**		
Believe natural is always safer	0.65 (0.14)	0.53 (0.12)
Agree the public has a right to know about chemicals	0.67 (0.13)	0.45 (0.14)
Feel able to find information about safety of chemicals	0.92 (0.05)	0.72 (0.13)
Would stop using a product containing unsafe chemical	1.00 (< 0.01)	0.69 (0.15)
Agree that products ought to be tested fully to ensure safety	Not estimable	0.69 (0.15)
Read the label on products	0.89 (0.08)	0.55 (0.12)
Think about risks of products, prior to purchase	0.52 (0.14)	0.32 (0.11)
Trust that a store will only sell safe products	0.66 (0.13)	0.41 (0.12)

Table S3. Twin polychoric correlation (and standard error) in item responses among white participants, by twin pair type. Missing data were handled by pairwise deletion. Correlations are on scale of observed responses, not as recoded for analysis, except # of fragranced products (recoded "I don't know" as missing).

	Identical	Fraternal	Same Address	Different Addresses
**Frequency of Use of Fragranced Products**				
Shampoo or conditioner	0.66 (0.09)	0.38 (0.22)	0.68 (0.12)	0.57 (0.12)
Shower soap or body wash	0.43 (0.12)	0.46 (0.18)	0.68 (0.15)	0.26 (0.14)
Hand soap	0.37 (0.11)	0.30 (0.25)	0.49 (0.13)	0.27 (0.14)
Body lotion	0.44 (0.10)	0.19 (0.29)	0.66 (0.11)	0.23 (0.13)
Perfume or cologne	0.65 (0.06)	0.45 (0.25)	0.71 (0.07)	0.50 (0.11)
Laundry detergent	0.48 (0.11)	0.36 (0.30)	0.64 (0.11)	0.41 (0.14)
Bathroom or kitchen cleaners	0.55 (0.06)	0.10 (0.19)	0.48 (0.15)	0.46 (0.08)
Floor polish	0.30 (0.13)	0.79 (0.17)	0.61 (0.17)	0.21 (0.16)
Furniture polish	0.46 (0.10)	0.82 (0.10)	0.68 (0.10)	0.36 (0.13)
# different fragranced products used per week	0.23 (0.13)	0.11 (0.30)	0.21 (0.19)	0.22 (0.16)
**Locations of Use of Air Fresheners**				
Use in bathroom	0.34 (0.14)	0.57 (0.32)	0.30 (0.21)	0.35 (0.16)
Use in bedroom	0.36 (0.16)	−0.20 (0.39)	0.50 (0.19)	0.04 (0.22)
Use in laundry room	0.30 (0.25)	Not estimable	Not estimable	0.55 (0.24)
Use in living room / den	0.66 (0.11)	0.24 (0.44)	0.86 (0.10)	0.40 (0.16)
Use in basement	0.57 (0.23)	Not estimable	Not estimable	0.60 (0.24)
Use in car	0.60 (0.11)	0.22 (0.36)	0.81 (0.11)	0.37 (0.17)
Never use air fresheners	0.55 (0.14)	0.15 (0.48)	0.47 (0.22)	0.49 (0.17)
**"Have you [ever] heard…"**				
"…that some plastic bottles may be unsafe to drink from, or heard of BPA (bisphenol A)?"	0.72 (0.12)	Not estimable	0.76 (0.15)	0.37 (0.26)
"…of lead, or thought about lead exposure?"	0.52 (0.14)	0.34 (0.37)	0.64 (0.17)	0.40 (0.19)
"…of mercury or thought about mercury exposure?"	0.18 (0.18)	Not estimable	0.22 (0.26)	0.42 (0.19)
"…of parabens or thought about exposure to parabens?"	0.77 (0.10)	Not estimable	0.67 (0.18)	0.67 (0.15)

Table S3. *Cont.*

	Identical	Fraternal	Same Address	Different Addresses
**Chemical Sensibilities**				
Get bothered by fragrances	0.49 (0.10)	0.16 (0.31)	0.63 (0.15)	0.34 (0.12)
Feel unwell after smelling fragrances in products	0.61 (0.09)	0.58 (0.23)	0.71 (0.13)	0.52 (0.11)
Prefer to buy organic produce	0.68 (0.10)	0.09 (0.37)	0.73 (0.13)	0.56 (0.13)
**Attitudes Concerning Chemical Safety**				
Believe natural is always safer	0.27 (0.13)	0.35 (0.19)	0.22 (0.17)	0.31 (0.13)
Agree the public has a right to know about chemicals	0.23 (0.13)	0.85 (0.16)	0.29 (0.21)	0.37 (0.14)
Feel able to find information about safety of chemicals	0.43 (0.12)	0.12 (0.30)	0.61 (0.15)	0.26 (0.16)
Would stop using a product containing unsafe chemical	0.25 (0.14)	−0.05 (0.37)	0.24 (0.20)	0.27 (0.16)
Agree that products ought to be tested fully to ensure	0.47 (0.11)	0.18 (0.37)	0.61 (0.15)	0.43 (0.14)
Read the label on products	0.46 (0.10)	0.35 (0.25)	0.45 (0.16)	0.44 (0.11)
Think about risks of products, prior to purchase	0.43 (0.09)	0.75 (0.14)	0.57 (0.13)	0.44 (0.11)
Trust that a store will only sell safe products	0.19 (0.12)	0.38 (0.22)	0.32 (0.15)	0.15 (0.14)

Table S4. Pairwise approximate polychoric correlations and standard errors of indicators of attitudes toward chemical safety among black or white participants. Missing data were handled by pairwise deletion. "Approximate" because correlations and standard errors ignore twin clustering.

**1**	**Believe Natural is Always Safer**							
0.30 (0.08)	1	Agree the public has a right to know about chemicals						
0.03 (0.08)	0.14 (0.08)	1	Feel able to find information about safety of chemicals					
0.27 (0.08)	0.30 (0.08)	0.04 (0.08)	1	Would stop using a product containing unsafe chemical				
0.29 (0.08)	0.57 (0.08)	0.02 (0.08)	0.38 (0.07)	1	Agree that products ought to be tested fully to ensure safety			
0.13 (0.08)	0.37 (0.08)	0.25 (0.07)	0.02 (0.08)	0.01 (0.09)	1	Read the label on products		
0.21 (0.07)	0.30 (0.07)	0.14 (0.07)	0.27 (0.07)	0.13 (0.08)	0.57 (0.05)	1	Think about risks of products, prior to purchase	
−0.14 (0.07)	−0.24 (0.08)	−0.04 (0.08)	−0.05 (0.08)	−0.08 (0.08)	−0.14 (0.08)	−0.37 (0.06)	1	Trusts that store will only sell safe products

Table S5. Self-Reported Frequency of Use of Fragranced Products. Black and white participants are aggregated (*N* = 314); data are presented on scale of analysis (collapsed categories from self-report).

Product	Frequency	N
Shampoo	Weekly or less	63
	Several times a week	90
	Daily or more	156
Body Wash	Weekly or less	55
	Several times a week	69
	Daily or more	189
Body Lotion	Never	88
	Less than daily	122
	Once or more per day	102
Handsoap	Once a week or less	63
	Several times a week to daily	79
	More than once per day	169
Laundry Detergent	Less than once per week	87
	Once per week	91
	More than once per week	133
Cleaners	Less than once per week	101
	Once per week	57
	More than once per week	154
Furniture Polish	Never or almost never	169
	Less than once per week	84
	More than once per week	57
Floor Polish	Never use	209
	Ever use	64
Perfume	Never use	88
	Use less than daily	119
	Once or more daily	103
How Many / Week	None	10
	1–3 different products	134
	4–6 different products	120
	7 or more different products	42

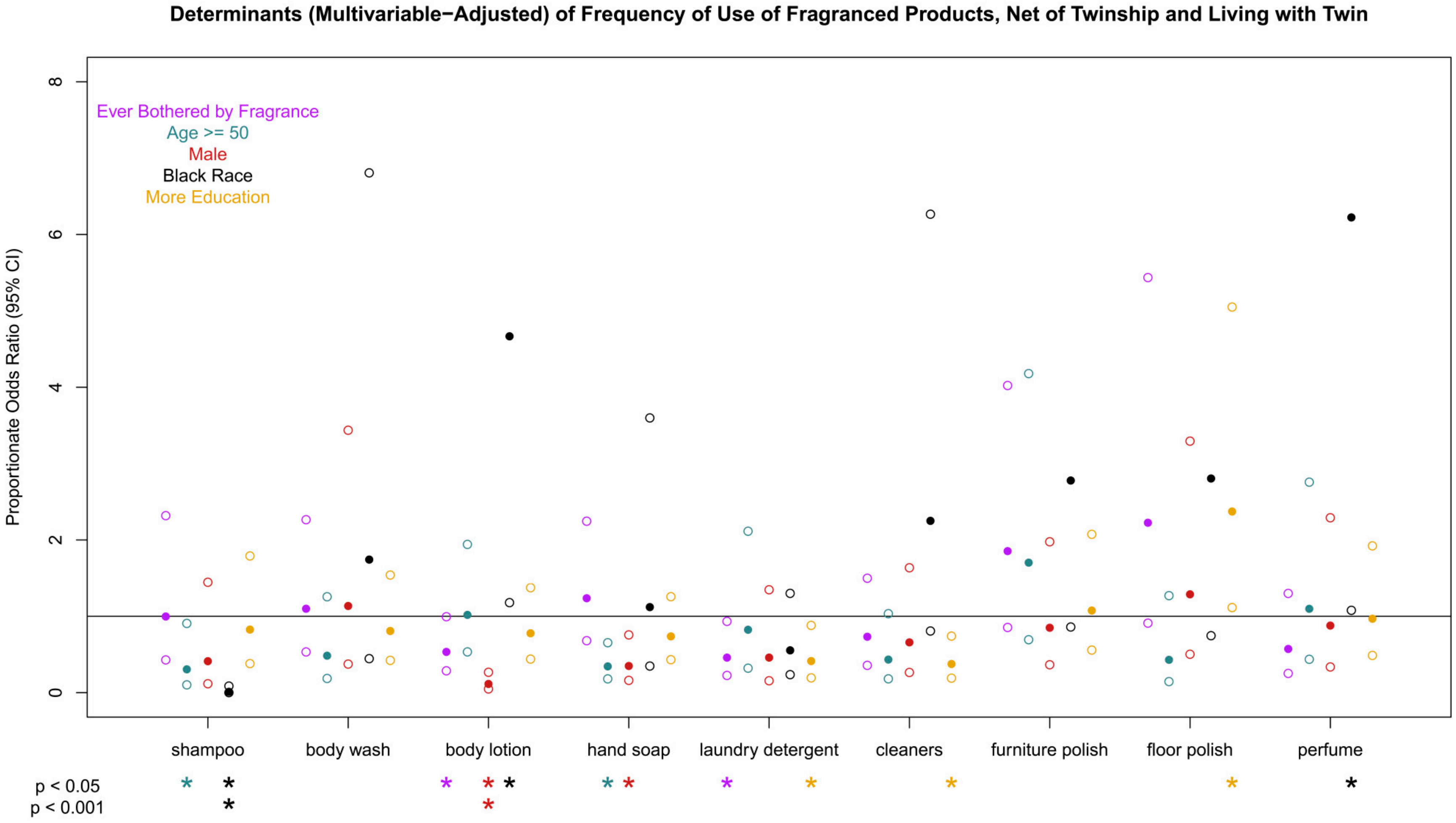

Figure S1. Demographic predictors of fragranced product use (multivariable-adjusted). Models are ordered logistic regressions with random intercepts for twin pair and address, fit jointly for all demographic characteristics. Only the proportionate odds ratio estimate for black race includes black participants ($N = 314$); other estimates are from models with white participants only ($N = 280$). Missing data were imputed by multiple imputation with chained equations. Filled circles are point estimates, empty circles are 95% confidence intervals. If confidence interval upper bound > 8, not shown.

Table S6. Principal components of self-reported fragranced product use among white participants. Missing data were handled by listwise deletion (*N* = 242).

	PC1	PC2	PC3	PC4	PC5	PC6	PC7	PC8	PC9
% Explained	0.30	0.16	0.11	0.10	0.09	0.07	0.07	0.05	0.05
Cumulative % Explained	0.30	0.46	0.57	0.67	0.76	0.83	0.90	0.95	1.00
Product Use Loadings									
Shampoo or conditioner	0.23	0.45	−0.18	−0.06	0.71	0.21	0.31	0.26	−0.07
Shower soap or body wash	0.36	0.38	0.20	−0.17	0.16	−0.13	−0.67	−0.40	−0.02
Hand soap	0.35	0.13	0.07	−0.59	−0.43	0.43	0.36	0.12	0.33
Body lotion	0.35	0.15	0.55	0.06	−0.17	−0.53	0.16	0.23	−0.23
Perfume or cologne	0.29	0.16	0.15	0.77	−0.18	0.44	0.02	−0.08	0.17
Laundry detergent	0.38	−0.04	−0.55	0.15	−0.15	−0.33	−0.31	0.51	0.20
Bathroom or kitchen cleaners	0.41	−0.13	−0.47	−0.01	−0.10	−0.12	0.37	−0.60	−0.26
Floor polish	0.27	−0.54	0.23	−0.01	0.43	−0.13	0.09	−0.13	0.60
Furniture polish	0.32	−0.52	0.14	−0.06	0.11	0.36	−0.23	0.24	−0.59

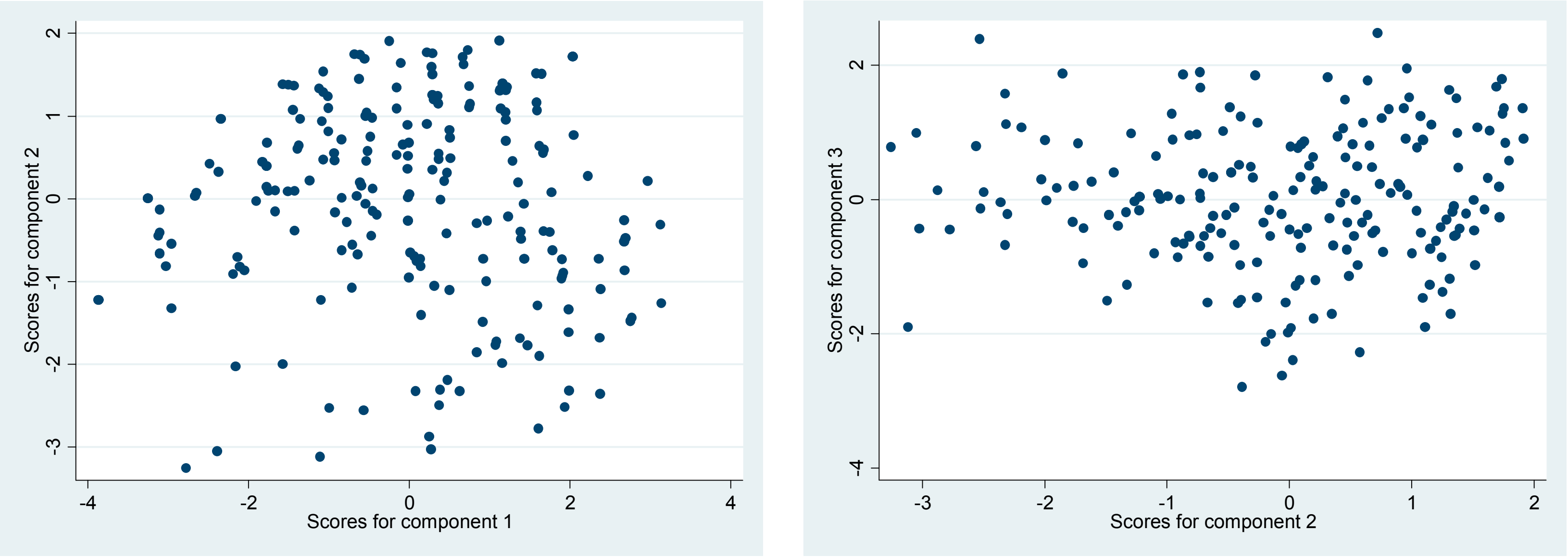

(**a**) (**b**)

Figure S2. Plots of the first 3 principal components of fragranced product use among white participants. Panel (**a**): principal component 1 vs. principal component 2. Panel (**b**): principal component 2 vs. principal component 3.

© 2015 by the authors; licensee MDPI, Basel, Switzerland. This article is an open access article distributed under the terms and conditions of the Creative Commons Attribution license (http://creativecommons.org/licenses/by/4.0/).
